# Synthesis, Activation, and Characterization of Carbon Fiber Precursor Derived from Jute Fiber

**DOI:** 10.1021/acsomega.4c01268

**Published:** 2024-08-05

**Authors:** Md Shahabul Hossen, Tarikul Islam, Sheikh Manjura Hoque, Aminul Islam, M. Mahbubul Bashar, Gajanan Bhat

**Affiliations:** † Department of Textile Engineering, Mawlana Bhashani Science and Technology University, Tangail, Santosh 1902, Bangladesh; ‡ Department of Textiles, Merchandising, and Interiors, University of Georgia, Athens, Georgia 30602, United States; § Department of Textile Engineering, Jashore University of Science and Technology, Jashore 7408, Bangladesh; ∥ Materials Science Division, Bangladesh Atomic Energy Commission, Atomic Energy Centre, Dhaka 1000, Bangladesh; ⊥ Department of Petroleum and Mining Engineering, Jashore University of Science and Technology, Jashore 7408, Bangladesh

## Abstract

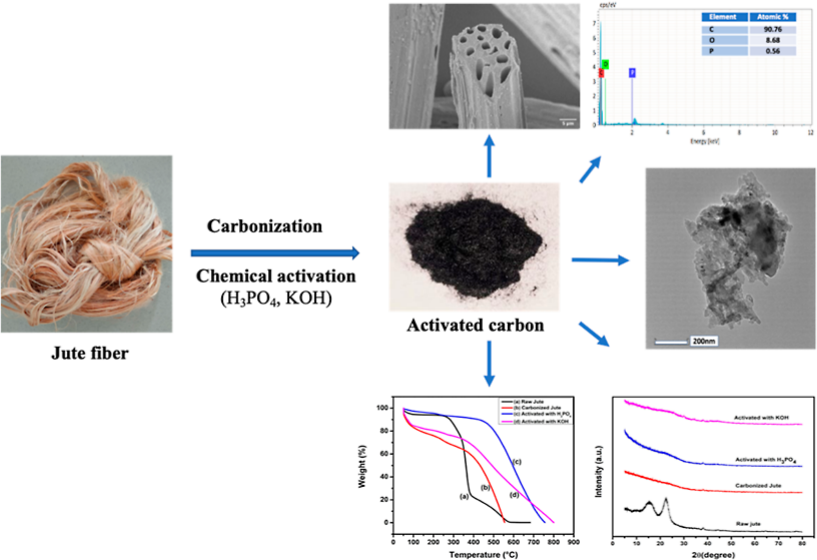

Activated carbon (AC) fiber is a carbonaceous material with a porous structure that has a tremendous scope of application in different fields. Conventionally, AC is derived from fossil fuel-based raw materials like polyacrylonitrile (PAN) and pitch. In this work, AC was synthesized from eco-friendly, renewable, and ubiquitous jute fiber. Systematically, the jute fiber was washed and pretreated with NaOH. Raw jute and NaOH-treated jute were carbonized/pyrolyzed at 500 °C for 1 h in presence of N_2_ gas. The carbonized carbon was activated with H_3_PO_4_ and KOH and again pyrolyzed at 650 °C for 1.5 h maintaining the inert condition. The different features of activated carbons were characterized with field emission-scanning electron microscope, energy-dispersive X-ray spectroscopy (EDX), transmission electron microscopy (TEM), X-ray diffraction (XRD), and thermogravimetric analysis. The average yield of carbonized and activated carbons was recorded at 19 and 13.8%, respectively. The scanning electron microscopic images confirmed a honeycomb-like porous structure. It was observed that KOH-activated carbon exhibited a more porous structure than the H_3_PO_4_-activated carbons. The average pore diameter of activated carbons was noted to be 1.3 μm. The pore density was higher in case of KOH-activated carbons accounting for 2.15 pore/μm. The EDX analysis showed that H_3_PO_4_-activated carbons had more than 90% carbon atoms indicating a significant carbon content. The TEM images revealed that AC particles were in the nanoscale range. The average particle sizes of H_3_PO_4_-activated carbon and KOH-activated carbon were 36.38 and 32.8 nm, respectively. The XRD study demonstrated the highly disordered and low level of crystallinity of AC. It was detected that the AC showed much higher thermal resistance than the jute fiber. The H_3_PO_4_-activated carbon obtained from NaOH-treated jute remained at 84% even after 500 °C. A higher thermal resistance was achieved with H_3_PO_4_-activated carbon since it contains 0.56% phosphorus, which was confirmed by EDX investigation. It was found that a higher carbon yield was obtained from NaOH-treated jute. The porous structure of the material showed that it could be used as an adsorbent. Due to its high thermal stability, it is recommended for flame retardants and heat insulation applications as well.

## 1 Introduction

Activated carbon (AC) is a common form of carbon with a porous structure and high surface area.^1^ The term ‘AC’ refers to carbon with an active surface that can adsorb molecules like heavy metal ions, microbes, organic matter, etc. In recent years, AC has become widely used as an adsorbent material. The porous properties of AC make it an effective purifier of air (removing odors and toxic substances) and water (removal of minerals and organic matter).^2,3^ For example, it has been used to remove heavy metals such as chromium, metal arsenic, and pharmaceuticals from aqueous solutions,^4,5^ adsorb greenhouse gases and CO_2_ from the air,^6−8^ and remove dyes and coloring agents such as methylene blue from textile effluent.^2,8,9^ Besides being used as an adsorbent, it is also applied to make electronic material and catalyst support, for example, electrodes for supercapacitors and anodes for lithium-ion batteries which have outstanding cyclic stability.^10−14^ A supercapacitor made of AC retains 93% capacitance over 5000 cycles indicating greater energy density and durability.^15^

Traditionally, AC fibers are synthesized from fossil fuel-based raw material polyacrylonitrile (PAN).^16^ PAN is a well-known and popular precursor to carbon fiber (CF). PAN-based CF has a higher strength than other precursors. It produces high-performance CF because of its higher strength, higher carbon yield, superior melting point, and rapid pyrolysis process. Furthermore, its high carbon yield makes it more thermally stable.^17^ In addition to PAN, viscose rayon (regenerated cellulose),^18^ petroleum coke,^19^ and phenolic resin^20^ are also used. Even though they have excellent properties, they are nonrenewable, expensive, and release hazardous gases like CH_4_, CO_2_, CO, NH_4_, and HCN.^21−23^ In order to overcome the issue of raw materials, researchers are focusing on finding renewable and sustainable alternatives from natural fibers.^24^ In this regard, natural sources such as biomass can be a promising candidate for AC, as it is a great source of lignocellulose and is renewable, eco-friendly, and sustainable. Therefore, scientific efforts are currently focusing on biomass as a raw material for AC.^25^ In recent years, a wide range of biomasses has been explored to manufacture AC, including tea waste,^26^ fruit peel,^27^ grapefruit peel,^28^ pine cones,^29,30^ tobacco rods,^31^ aloe vera,^32^ jute sticks,^33^ rice husk,^34^ coffee husks,^22,35^ almond shells,^36^ bamboo sawdust,^37^ coconut,^9^ sugar beets,^38^ dipterocarpus alatus fruit,^2^ and corn cobs,^39^ corn husk,^40^ etc. However, most biomass shows scarcity and is not economically profitable. Therefore, selecting the proper biomass is still a challenge. In this context, jute is ubiquitous, cost-effective, and sustainable as well as easy to grow, requires less fertilizer and pesticides for cultivation, and has a short life cycle. In addition, jute fiber is highly potential source of activated carbons in terms of the comparatively high content of α-cellulose (more than 70%) next to the highest source of α-cellulose in cotton fiber,^41^ economic importance due to the high production volume of approximately 3 million tons per year,^42^ and the eco-friendly life cycle approach of jute cultivation. Considering the cellulose content, cotton fiber is the ultimate choice, as it is the highest and purest form of cellulose. Apart from this, cotton cultivation consumes enormous amounts of synthetic fertilizers and pesticides and exploits huge amounts of water, which are considered harmful and have pollution-loaded footprints for the world. On the contrary, jute fiber cultivation boosts the reduction of greenhouse gases like CO_2_. The statistics revealed that a hectare of jute plants consumed 15 tons of carbon dioxide and released 11 tons of oxygen.^43^ By rotation cultivation of jute plants further increases the fertility of the land. Hence, the jute plant is a kind of environmentally conserving plant, and its fiber is a pure green and sustainable source of AC.

AC synthesis includes carbonization followed by an activation process.^22^ In the carbonization process, raw materials are thermally decomposed under inert gases such as N_2_ and argon. It removes noncarbonaceous elements such as H_2_, steam, O_2_, etc.^44^ Carbonized carbon can be activated in two ways: physical and chemical activation. The physical activation method involves subjecting the carbonized carbon to high temperatures (600–900 °C) in the presence of an oxidizing agent, such as CO_2_, N_2_, steam, or a combination of these.^45^ This process does not involve any chemicals; therefore, it has a lower cost. However, it has a few drawbacks, such as low carbon yield,^46^ the release of greenhouse gases like CH_4_ and CO_2_,^22,47^ low adsorption capacity, and high energy consumption.^36^ The chemical activation procedure involves impregnating the carbonized carbon in an activating agent (such as a base, acid, or alkali) and followed by heating with high temperatures ranging from 500 to 700 °C.^48,49^ A variety of activating agents have been used in recent studies including H_3_PO_4_,^48,50^ KOH,^51^ CuCl_2_,^52^ HNO_3_,^53^ AlCl_3_, NH_4_Cl,^18^ and K_2_CO_3_.^54^ Chemical activation is preferable because its activation temperature is low, porous structures can be developed,^55^ it is eco-friendly, and it saves energy and time.^36^ In addition, chemically AC has a high surface area and a high adsorption capacity.^55,56^

As a biomass, jute is an ideal candidate for AC synthesis. Though there are few studies of AC synthesis from jute fiber, the thermal stability and particle size with H_3_PO_4_ activation^48,51,57−59^ are still unexplored. In this current study we have pretreated raw jute with NaOH for superior outcome as alkali treatment enhanced the cellulose content.^60^ Therefore, we attempted to synthesize AC from sustainable, renewable, and eco-friendly source of jute fiber and investigated the thermal stability, particle size, and percentages of carbon atom to fulfill the knowledge gap of the scientific community. Consequently, this research explores the synthesis of porous activated carbons from raw jute and NaOH-treated jute fiber, which could be a suitable candidate as an adsorbent as well as material for high thermal insulation applications.

## 2 Experimental Section

### 2.1 Materials

The jute fiber used in this study was collected from a local market of Bangladesh. Sodium hydroxide (NaOH pellet form, extra pure 98%), potassium hydroxide (KOH pellet form, extra pure 85%), and phosphoric acid (H_3_PO_4_(l), extra pure 85%) were purchased from a company located in Dhaka, Bangladesh.

### 2.2 Synthesis of AC

**Figure 1:**
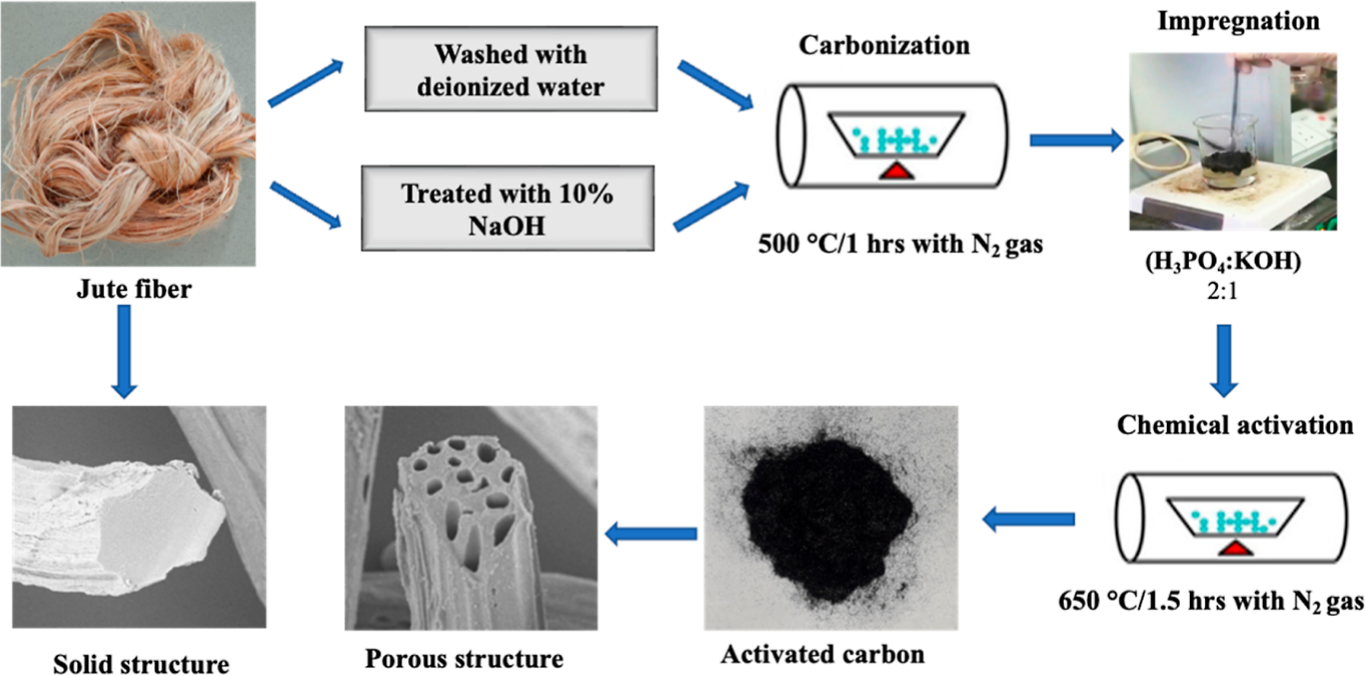
Schematic of synthesis process of AC from jute fiber.

First, raw jute was washed with deionized water to remove impurities and dried in an oven at 105 °C for 12 h. Second, the raw jute was treated with 10% NaOH solution for 3 h at 20 °C, washed with deionized water and dried. This caustic treatment of jute removed the hemicellulose and lignin, thus, enhancing the cellulose content of the jute fiber. The jute fiber was carbonized at 500 °C for 1 h with a 5 °C/min heating rate with a continuous N_2_ gas flow in a tube furnace (SANTE FURNACE, SAF-Therm). The following process was chemical impregnation, where carbonized jute was impregnated with an activating agent, such as acid or alkali. Here, carbonized samples were impregnated with KOH and H_3_PO_4_ with an impregnation ratio of 2:1. The chemical impregnation ratio refers to the ratio between the mass of the activating agent and the mass of the raw material. Afterward, samples were washed with deionized water and dried overnight in an oven at 100 °C. The impregnated samples were pyrolyzed again in an inert environment with a nitrogen gas flow, which is known as chemical activation. The material was carbonized at 650 °C for 90 min with a heating rate of 7 °C/min and thus produced AC. An overview of the synthesis of AC from jute fiber is shown in Figure 1.

### 2.3 Characterization

The cross section and porous structure were studied with field emission scanning electron microscopy (FE-SEM, ZEISS Sigma 300). The elemental compositions of all samples were determined by using EDX. The EDX was connected to the FE-SEM. A transmission electron microscope was used to observe the particle size and surface morphology. TEM images were captured by a ThermoFisher Scientific transmission electron microscope (the model is Talos, F_200_X G_2_, serial number 9951137, 2018, manufactured in the Czech Republic). The TEM magnification was 120 KX for carbonized carbon, 58 KX for H_3_PO4-activated carbon, and KOH-activated carbon. Different particle sizes were measured from the TEM images to create the TEM graphs. The samples were immersed in ethanol and sonicated for several minutes for TEM observation. The crystal structure of all samples was examined with an X-ray diffractometer (SmartLab, Japan). The X-ray diffractometer was operated at 40 mA power and 40 kV voltage, and a Cu-kα X-ray source was applied to generate the XRD pattern. The crystallinity index (CI) was calculated based on eq 1.
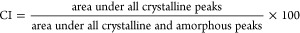
1

Thermal stability was investigated by using TGA. The test was performed with a TGA 4000 instrument (Brand: Parkin Elmer). The experiment was conducted at 50 to 800 °C at a heating rate of 10 °C/min under N_2_ gas flow of 20 mL/min to prevent material combustion.

## 3 Results and Discussion

### 3.1 Surface Morphology

**Figure 2:**
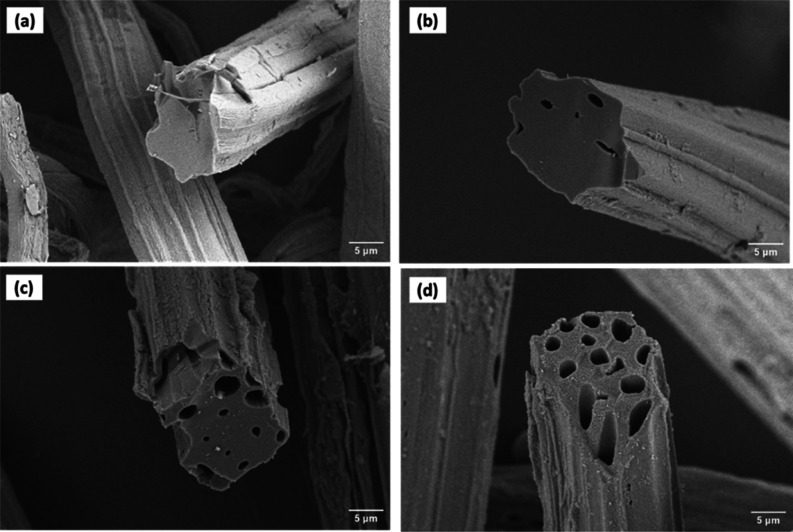
SEM micrographs of (a) raw jute fiber, (b) carbonized jute, (c) activated with H_3_PO_4_, and (d) activated with KOH.

**Figure 3:**
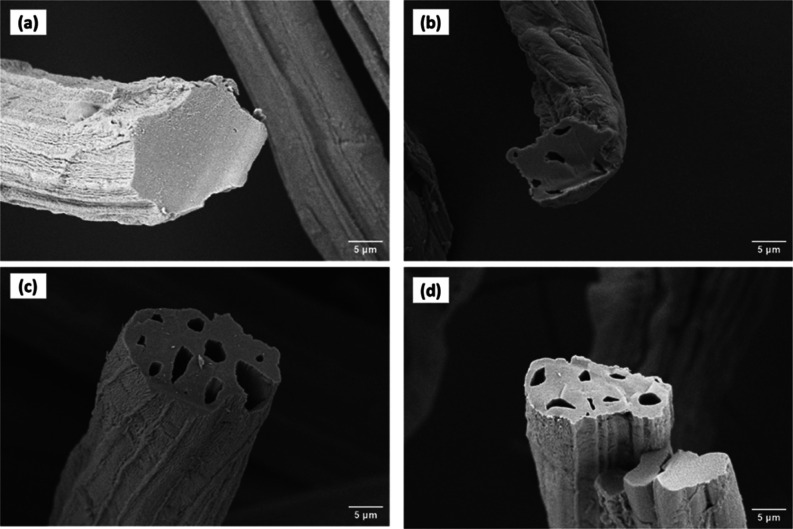
SEM micrographs of (a) NaOH-treated jute, (b) carbonized NaOH-treated jute, (c) activated NaOH-treated jute (H_3_PO_4_), (d) activated NaOH-treated jute (KOH).

The SEM micrographs of raw jute, NaOH-treated jute, carbonized carbon, and AC derived from untreated jute and NaOH-treated jute are shown in Figures 2 and 3, respectively. Figures 2a and 3a show the surface morphology and cross section of raw jute fiber and NaOH-treated fiber, respectively. It reveals a solid and rod-like shape without any porous structure in the cross section. Figures 2b and 3b demonstrate that few pores were created after carbonization. However, the number of pores was inadequate, and most were blocked. Non-carbonaceous elements like O_2_, H_2_, and steam were removed here.^35^Figures 2c,d, and 3c,d represent images of carbon activated with H_3_PO_4_ and KOH. The general mechanism of activating carbon with chemical agents such as H_3_PO_4_ and KOH relies on the destruction of cellulose structures followed by char formation and aromatization of the carbon skeleton.^62^ It was observed that a honeycomb-like porous structure was created after chemical activation. The blocked pores in carbonized carbon were opened after chemical activation.^48^ It was also found that KOH-activated carbon had wider pores than H_3_PO_4_-activated carbon, which was relevant to recent work.^63^ Since KOH is a strong base, it attracted the cell wall more vigorously than H_3_PO_4_ and got impregnated with carbon more quickly.^64,65^ Furthermore, the metallic potassium interacted with the cellulose cell wall, extending the space between carbon atomic layers, which was the driving force for generating increased total pore volume.^62^ On the contrary, activated and carbonized carbon from NaOH-treated jute were noticed less porous than that of untreated jute. In pretreatment with NaOH, hemicellulose and lignin were removed, and the cross section became denser, resulting in a less porous structure.^66^

### 3.2 Elemental Analysis by EDX

**Figure 4:**
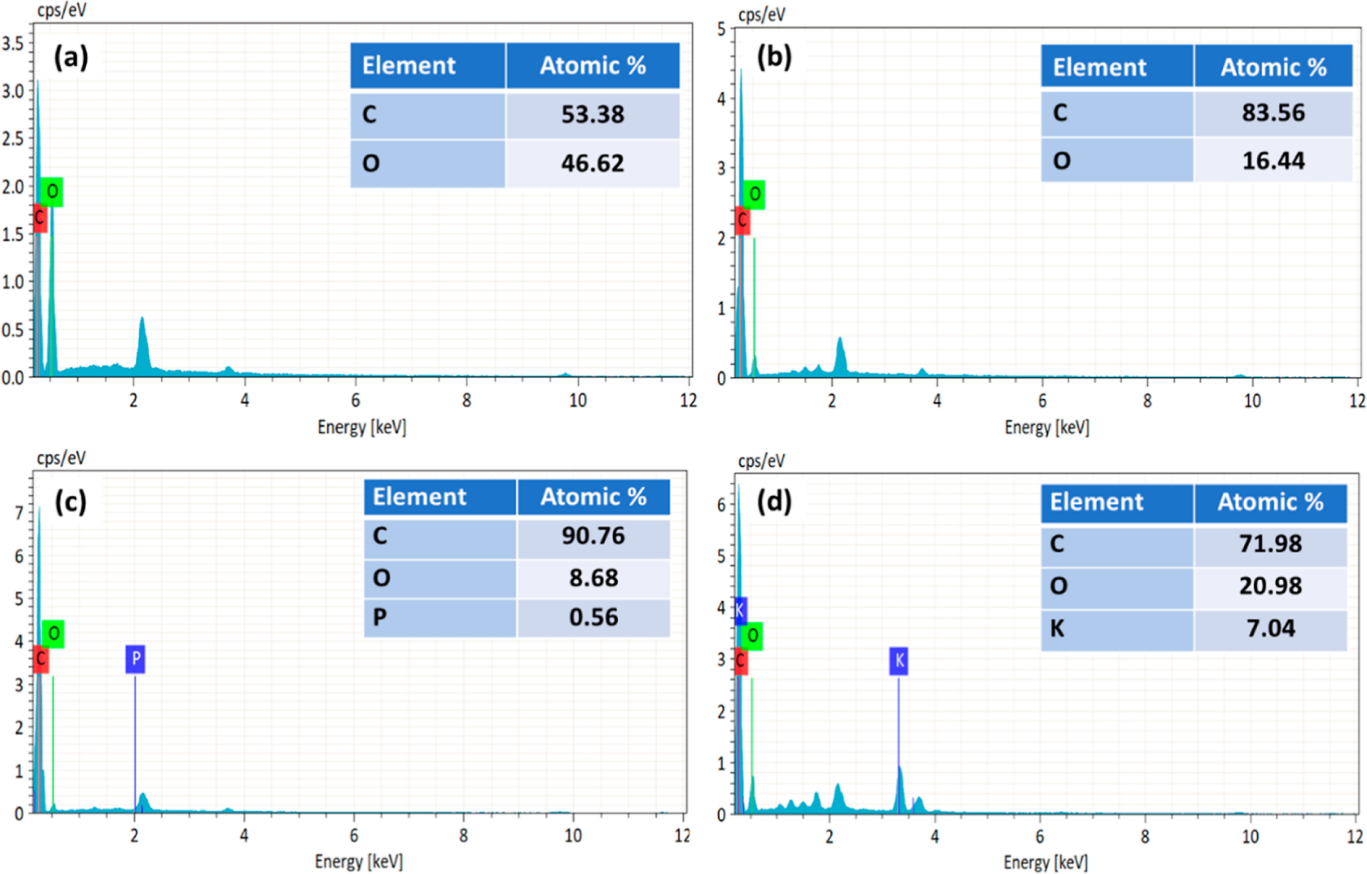
EDX spectra of (a) raw jute fiber, (b) carbonized jute, (c) activated with H_3_PO_4_, and (d) activated with KOH.

**Figure 5:**
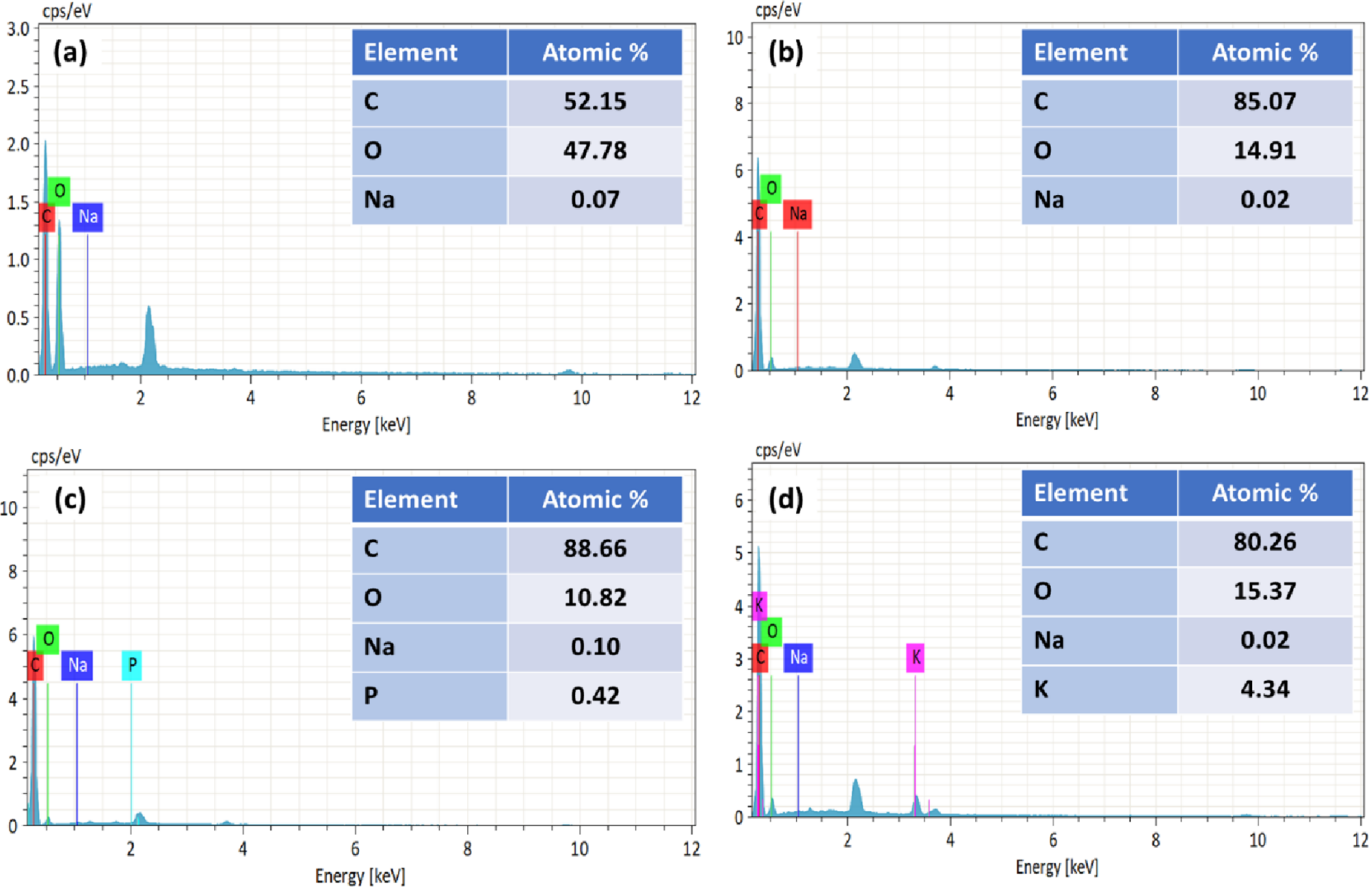
EDX spectra of (a) NaOH-treated jute, (b) carbonized NaOH-treated jute, (c) activated NaOH-treated jute (H_3_PO_4_), (d) activated NaOH-treated jute (KOH).

Figures 4 and 5 show the EDX profile of raw jute, carbonized carbon, and AC derived from untreated jute and NaOH-treated jute, respectively. The raw jute in Figure 4a and the NaOH-treated jute in Figure 5a demonstrated that the carbon content was lower. As a consequence of carbonization and activation, the carbon content was significantly increased by the removal of volatile materials such as steam, O_2_, and H_2_.^35^ The higher carbon content shown in Figure 4b, carbonized carbon (83.56%) and, shown in Figure 5b, NaOH-treated carbonized carbon (85.07%) indicated that the carbonization was successful.^36^ This outcome is supported by the previous studies.^48^ The AC showed a slightly higher carbon content than the carbonized carbon because the oxygen was partially decomposed due to the high temperature of the chemical activation process.^9^ As compared to H_3_PO_4_-activated carbon, KOH-activated carbon showed a lower carbon content. Since KOH is a strong alkali, it contains the (−OH) group that provided oxygen to AC, resulting in a lower carbon content.^65^

### 3.3 Particle Size Analysis by TEM

**Figure 6:**
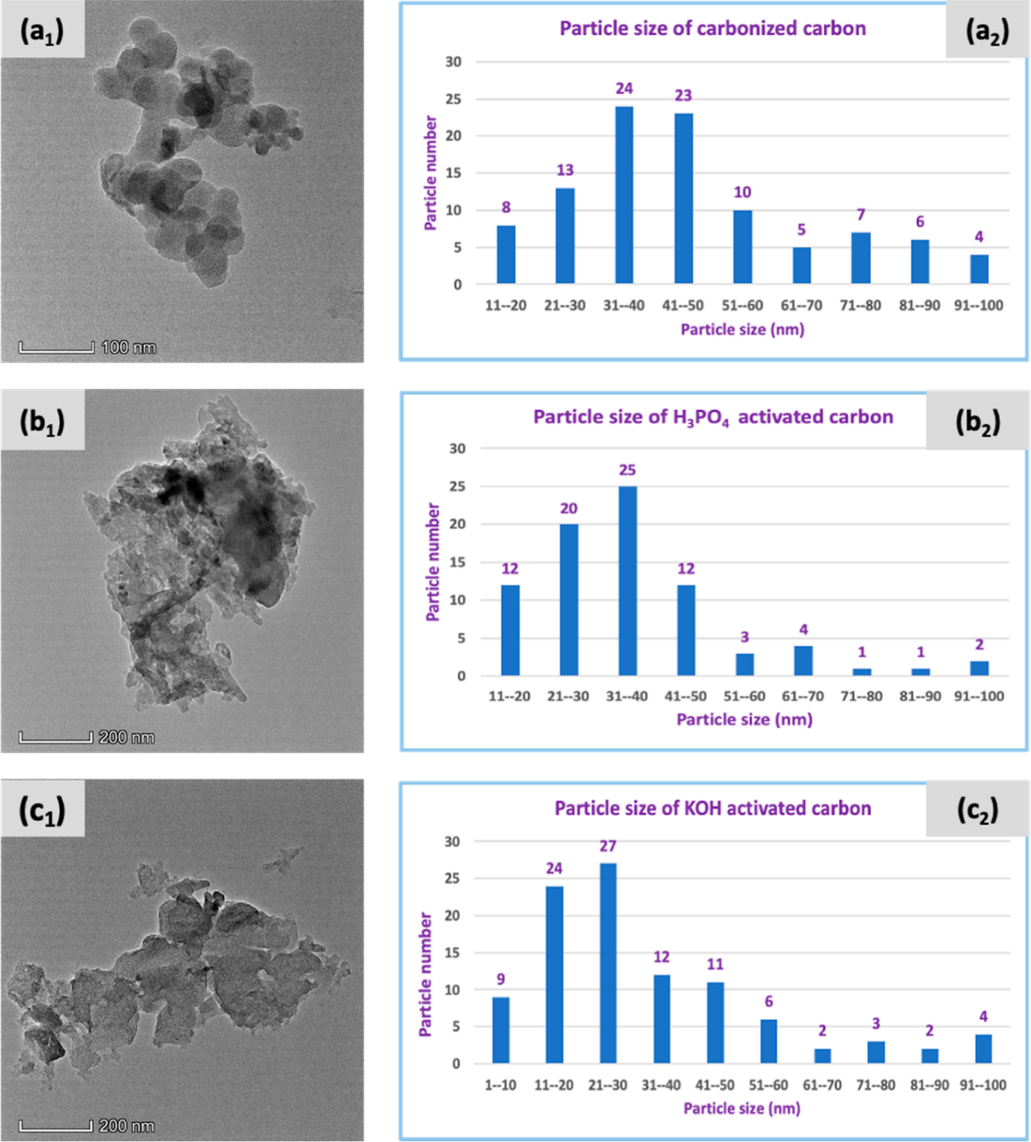
TEM images of (a_1_) carbonized NaOH-treated jute, (b_1_) activated NaOH-treated jute (H_3_PO_4_), and (c_1_) activated NaOH-treated jute (KOH) and graphs of particle sizes (a_2_) carbonized NaOH-treated jute, (b_2_) activated NaOH-treated jute (H_3_PO_4_), and (c_2_) activated NaOH-treated jute (KOH).

Figure 6 illustrates the TEM images of carbonized carbon and AC derived from raw jute fiber and NaOH-treated jute with a histogram of particle size. The TEM images depicted that particles were arranged randomly and overlapping one another. The particles of carbonized carbons appeared relatively round, whereas in AC the particles displayed rough and uneven shapes.^67^ The structure was amorphous because of the randomly arranged particles and a rough and uneven shape. The graph of carbonized carbon (a_2_) showed that most particles were found in the range of 21 to 60 nm. It was found that the majority of H_3_PO_4_-activated carbon particles ranged in size from 21 to 50 nm, while the maximum particles originating from KOH activation ranged in size from 11 to 50 nm. Table 1 shows the average particle sizes of carbonized carbon and AC. The average particle size of KOH-activated carbon was smaller since KOH interacted more vigorously with carbon and created more fractures in the carbon structure.

**Table 1:** Average Particle Size of Different Carbons

samples	avg. particle size (nm)
carbonized carbon	46.6
H_3_PO_4_-activated carbon	36.38
KOH-activated carbon	32.8

### 3.4 Crystal Structure Analysis by XRD

**Figure 7:**
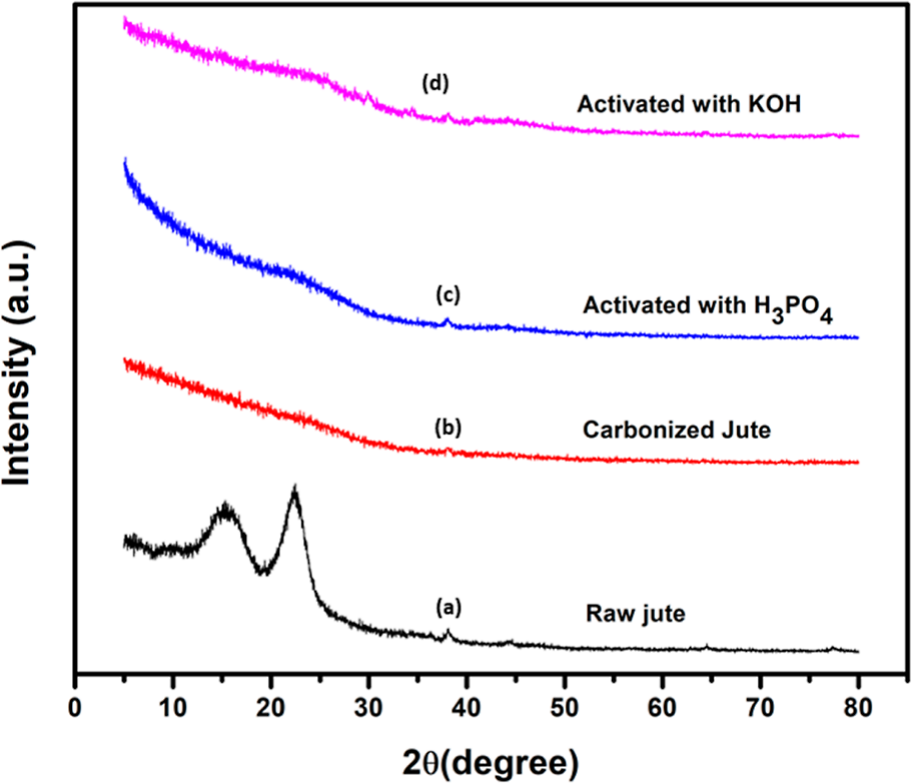
XRD patterns of (a) raw jute fiber, (b) carbonized jute, (c) activated with H_3_PO_4_, and (d) activated with KOH.

**Figure 8:**
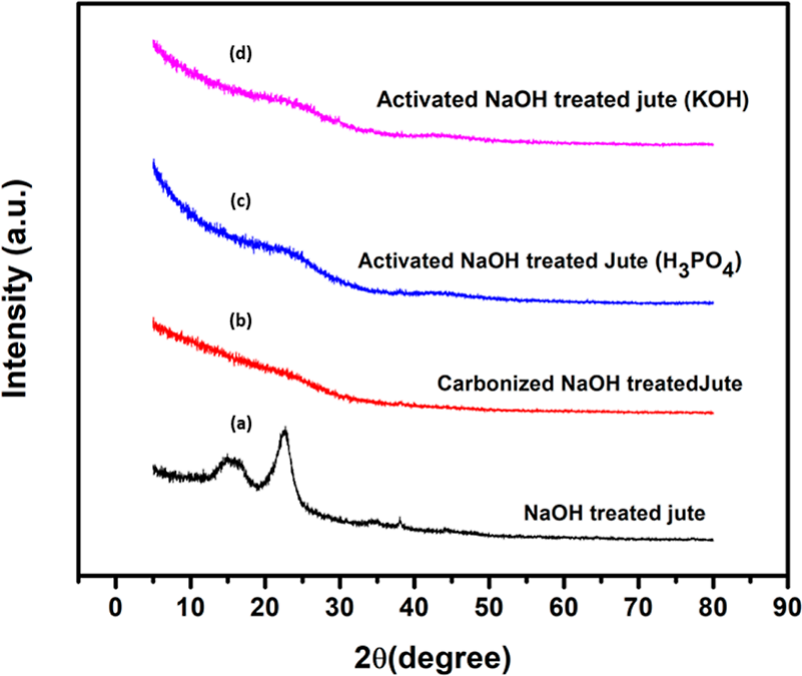
XRD patterns of (a) NaOH-treated jute, (b) carbonized NaOH-treated jute, (c) activated NaOH-treated jute (H_3_PO_4_), (d) activated NaOH-treated jute (KOH).

Figures 7 and 8 show the XRD patterns of raw jute, carbonized, and AC derived from raw jute and NaOH-treated jute, carbonized and AC, respectively. It was observed that raw jute fiber displayed two diffraction peaks at 2θ = 15.64° and 2θ = 22.46° attributed to cellulose I. On the contrary, the NaOH-treated jute fiber also exhibited two peaks at 2θ = 15.2° and 2θ = 22.68° for the cellulose II structure.^68^Table 2 demonstrates the CI of raw and NaOH-treated jute fiber. Interestingly, the NaOH treatment drastically improved the crystallinity, accounting for 71.11%, which was higher than that of raw jute fiber, resulting in 63.5%. This was due to removing non-cellulosic components like hemicellulose and lignin.^61^ The same outcome was reported by Camila Soares et al.^69^ It appeared that carbonized carbon derived from raw jute and NaOH-treated jute had a broad peak at 2θ = 20–30°, indicating the amorphous structure. All of the samples of AC exhibited a very broad peak at 2θ = 20–30° and a less prominent peak at 2θ = 43°. These broad, weak peaks revealed amorphous carbon. It was clear from this finding that the AC had poor graphitization.^51,70,71^Table 2 shows a lower CI for carbonized and activated carbons, suggesting highly amorphous structures. The activated carbons with KOH and phosphoric acid did not differ significantly in their amorphous structures.

**Table 2:** Crystallinity Index (%) of all Samples

samples	crystallinity Index (%)	samples	crystallinity index (%)
raw jute	63.50	NaOH-treated jute	71.11
carbonized jute	14.73	carbonized NaOH-treated jute	14.09
activated with H_3_PO_4_	7.73	activated NaOH-treated jute (H_3_PO_4_)	9.11
activated with KOH	12.04	activated NaOH-treated jute (KOH)	10.04

### 3.5 Thermal Stability Analysis by TGA

**Figure 9:**
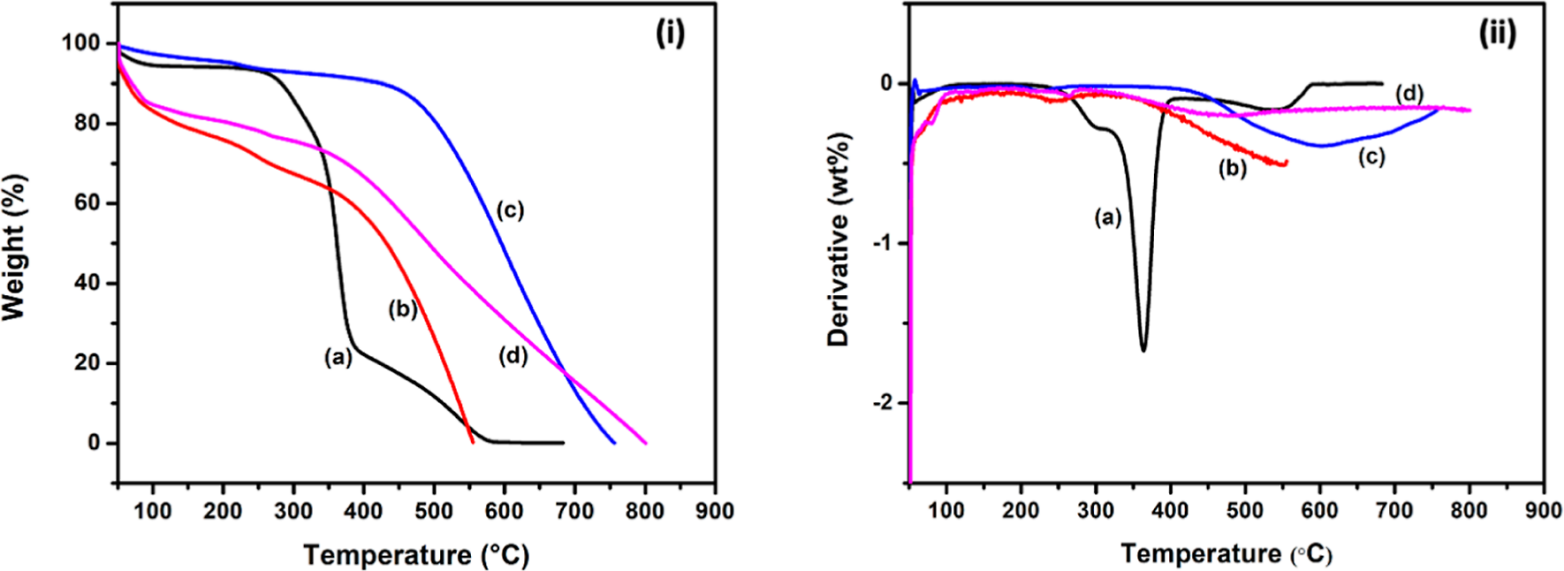
TGA (i) and DTG (ii) curves of (a) raw jute fiber, (b) carbonized jute, (c) activated with H_3_PO_4_, and (d) activated with KOH.

**Figure 10:**
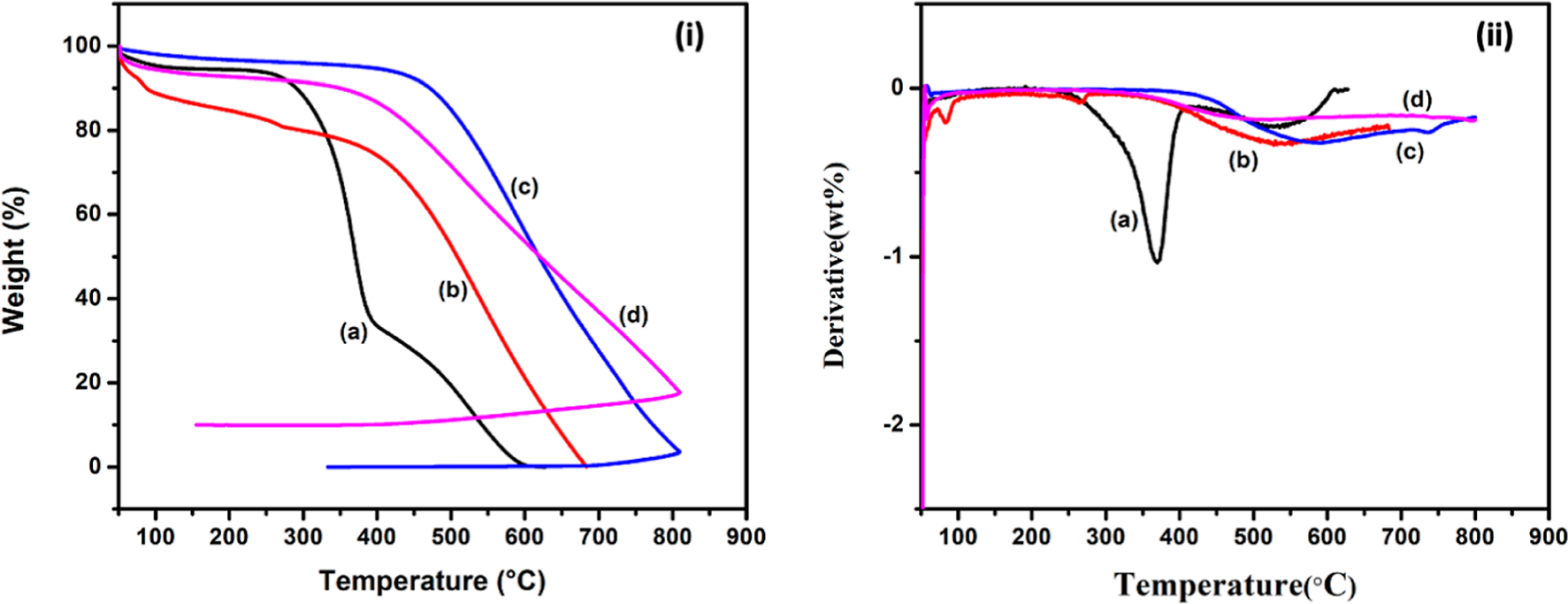
TGA (i) and DTG (ii) curves of (a) NaOH-treated jute, (b) carbonized NaOH-treated jute, (c) activated NaOH-treated jute (H_3_PO_4_), (d) activated NaOH-treated jute (KOH).

Figure 9 displays the TGA and differential thermogravimetry (DTG) profiles of raw jute (a), carbonized carbon (b), and AC (c,d) synthesized from untreated jute. In the same way, Figure 10 also shows the TGA and DTG profiles of NaOH-treated jute (a), carbonized carbon (b), and AC (c,d) produced from NaOH-treated jute. The DTG graph shows that the first degradation occurred for all samples at approximately 50 to 100 °C due to the removal of hygroscopic molecules.

It was noticed that raw jute was degraded in three stages. The hemicellulose degraded at 200 to 300 °C in the first stage, whereas cellulose degraded at 300 to 400 °C in the second stage. During the third stage, lignin was decomposed from 140 to 682 °C. These results are relevant to the TGA and DTG profiles discussed in the previous study.^30,72^ In NaOH-treated jute (see Figure 10i), the distinctive peak for hemicellulose did not appear, as the hemicellulose was mostly removed during NaOH pretreatment.^66^ The remaining hemicellulose and cellulose were decomposed between 200 and 400 °C. The degradation of lignin was observed up to 600 °C. The carbonized carbon derived from raw jute and NaOH-treated jute exhibited a miniature peak at 360–553 °C and 370–680 °C, respectively. As compared to raw jute, carbonized carbon exhibited more thermal stability as it had been devolatilized (removing water, CO_2_, CH_4_, etc.) [98], and hemicellulose, cellulose, and lignin had also been removed^72,73^

The weight loss of H_3_PO_4_-activated carbon produced from raw jute and NaOH-treated jute began at approximately 425 °C and continued slowly to 800 °C (see Figure 9i). In the case of KOH-activated carbon made from raw jute and NaOH-treated jute, a slight degradation was observed at 370 °C and continued slowly to 800 °C. The AC was more thermally stable than carbonized carbon. After chemical activation, AC contained a higher level of a stable form of carbon atom that was more thermally resistant.^72^ The elemental analysis illustrated previously confirmed that AC was mostly composed of carbon atoms, therefore indicating the authenticity of this higher thermal stability.

Tables 3 and 4 illustrate the weight remaining percentages at different temperatures. The H_3_PO_4_-activated carbon obtained from raw jute and NaOH-treated jute remained at 80 and 84% at 500 °C, respectively. KOH-activated carbon from raw jute remained at 48%, while AC from NaOH-treated jute remained at 71% at 500 °C. H_3_PO_4_-activated carbon exhibited a higher thermal resistance than KOH-activated carbon because it has a higher carbon content and also contains the phosphorus element that has flame retardant properties.^74^

**Table 3:** Weight Remaining Percentage

weight remaining percentage					
temperature	100 °C	200 °C	300 °C	400 °C	500 °C
raw jute	94	94	85	22	11
carbonized jute	83	75	67	56	26
activated with H_3_PO_4_	97	95	92	90	80
activated with KOH	84	80	75	66	48

**Table 4:** Weight Remaining Percentage

weight remaining percentage					
temperature	100 °C	200 °C	300 °C	400 °C	500 °C
NaOH-treated jute	95	94	88	33	19
carbonized NaOH-treated jute	88	84	80	74	52
activated NaOH-treated jute (H_3_PO_4_)	98	96	95	94	84
activated NaOH-treated jute (KOH)	94	92	91	86	71

The thermal stability of activated and carbonized carbon derived from NaOH-treated jute was slightly greater than that of untreated jute. The NaOH removes hemicellulose, lignin, and dense components of jute, preventing weight loss.^66^ Considering the results, it was evident that AC has excellent thermal stability.

### 3.6 Yield Percentage

Table 5 represents the yield % of carbonized carbon and AC. Carbonized carbon yield percentages were almost the same, according to the previous studies.^66^ Compared with H_3_PO_4_-activated carbon, KOH-activated carbon showed a lower yield after chemical activation. Since KOH is a potential activating agent, it created a more porous structure, resulting in a lower yield. AC has a similar yield percentage to other biomass.^65^ Alkali treatment of jute fiber increases carbon yields due to the removal of hemicellulose, lignin, and other impurities.^66^

**Table 5:** Yield % of Carbonized Carbon and AC

sample	yield % of carbonized carbon	yield % of activated carbon	
raw jute	19	H_3_PO_4_-activated carbon	13.81
		KOH-activated carbon	12.02
10% NaOH	21.6	H_3_PO_4_-activated carbon	14.51
		KOH-activated carbon	13.75

## 4 Conclusions

In this research, AC was synthesized from raw jute and NaOH-treated jute fiber using KOH and H_3_PO_4_ as activating agents by the chemical activation process at 650 °C for 1.5 h. This study explores the influence of activating agents on different properties of AC. The SEM images revealed that the KOH-activated carbon had higher porosity than the H_3_PO_4_-activated carbon. The EDX analysis showed that H_3_PO_4_-activated carbon had 88–90% carbon atoms, while KOH-activated carbon had 70–80%. According to the TEM images, rough and unevenly shaped particles were arranged randomly, which revealed amorphous structures. The average particle size of H_3_PO_4_-activated carbon was 36.38 nm while that of KOH-activated carbon was 32.8 nm. The XRD results demonstrated that both carbonized and AC were amorphous in their physical structure. The H_3_PO_4_-activated carbon showed greater thermal resistance than KOH-activated carbon. The AC derived from NaOH-treated jute exhibited better thermal stability retaining 84% at 500 °C and a greater yield of 14.51%. The higher stability of AC made it suitable for use as a flame- and heat-retardant material. In addition, the presence of amorphous and porous structures suggests that it could be an effective adsorbent for removing toxic materials from the environment such as heavy metals and organic dyes from textile effluent. The future perspective of this study should be to explore the carbon-activating precursors from jute fiber by novel reagents to improve green manufacturing and the adsorption capacity of various organic pollutants like textile dyes from textile effluent.

## Supporting Information Available

Comparison of the experimental results and the literature results; fiber diameters; fiber pore size distributions in H_3_PO_4_-activated carbons and KOH-activated carbons; and dye uptake (mg/g) and dye removal (%) of KOH-activated carbons (PDF)
