# Supplementary material for: Synthesis, Activation, and Characterization of Carbon Fiber Precursor Derived from Jute Fiber
Source: ACS Omega. 2024 Aug 5;9(33):35384–93. doi: 10.1021/acsomega.4c01268 (PMC11339993; doi:10.1021/acsomega.4c01268)
Supplement: Supplementary file 1 — ao4c01268_si_001.pdf [file ao4c01268_si_001.pdf]

Supporting Information

Synthesis, Activation, and Characterization of Carbon Fiber Precursor Derived from Jute Fiber

Md Shahabul Hossen[1], Tarikul Islam[2,3,*], Sheikh Manjura Hoque[4], Aminul Islam[5], M. Mahbubul Bashar[1,*], Gajanan Bhat[2]

[1]Department of Textile Engineering, Mawlana Bhashani Science and Technology University, Santosh, Tangail 1902, Bangladesh
[2]Department of Textiles, Merchandising, and Interiors, University of Georgia, Athens, Georgia 30602, United States
[3]Department of Textile Engineering, Jashore University of Science and Technology, Jashore 7408, Bangladesh
[4]Materials Science Division, Bangladesh Atomic Energy Commission, Atomic Energy Centre, Dhaka 1000, Bangladesh
[5]Department of Petroleum and Mining Engineering, Jashore University of Science and Technology, Jashore 7408, Bangladesh

***Corresponding Authors**

M Mahbubul Bashar

Email: bashar.te@mbstu.ac.bd

Tarikul Islam

Email: tarikul@uga.edu; mti@just.edu.bd

Total 4 pages

3 tables

1 figure

1 text

Table S1: Comparison of the experimental results and the literature results.

Properties	Experimental result	Literature result	References
Carbon content	90.76%	90.73%	1
Crystal structure	Amorphous	Amorphous	2
Yields of carbonized carbon	(19-21.6) %	24.3%	3
Yields of activated carbon	(13.81-14.51) %	13.5%, 18.8%	4,5

Table S1 describes the comparison of experimental results and the literature results. The activated carbon has a carbon content of 90.76%, closely related to prior research findings[1]. Dou Y et al. investigated the amorphous crystal structure of activated carbon, and our research acquired the same[2]. The yield of carbonized carbon and activated carbon is (19-21.6%) and (13.8%,14.51%), respectively, which is consistent with the research of Cho D *et. a.l*, Basta A *et. al.*, and Cazetta A *et. al*[3–5] Consequently, we can conclude that the study results were satisfied with the works of literature.

Table S2: Fiber diameters.

S.I.	Samples	Fiber diameter *(μm)*
1.	Raw jute fiber	13.1-15.3
2.	Carbonized jute	13.7-15.8
3.	Activated with H_3PO_4	13.0-16.3
4.	Activated with KOH	15.0-16.5
5.	NaOH treated jute	14.2-19.9
6.	Carbonized NaOH-treated jute	11.4-12.3
7.	Activated NaOH-treated jute (H_3PO_4)	12.1-15.5
8.	Activated NaOH-treated jute (KOH)	10.2-13.8

Table S2 displays the diameters of raw jute fibers, carbonized carbons, and activated carbons. There is a minor variation in diameter among these samples. Activated carbon derived from NaOH-treated jute exhibited less diameter due to the removal of hemicellulose, and lignin and became denser during pretreatment with NaOH[3].

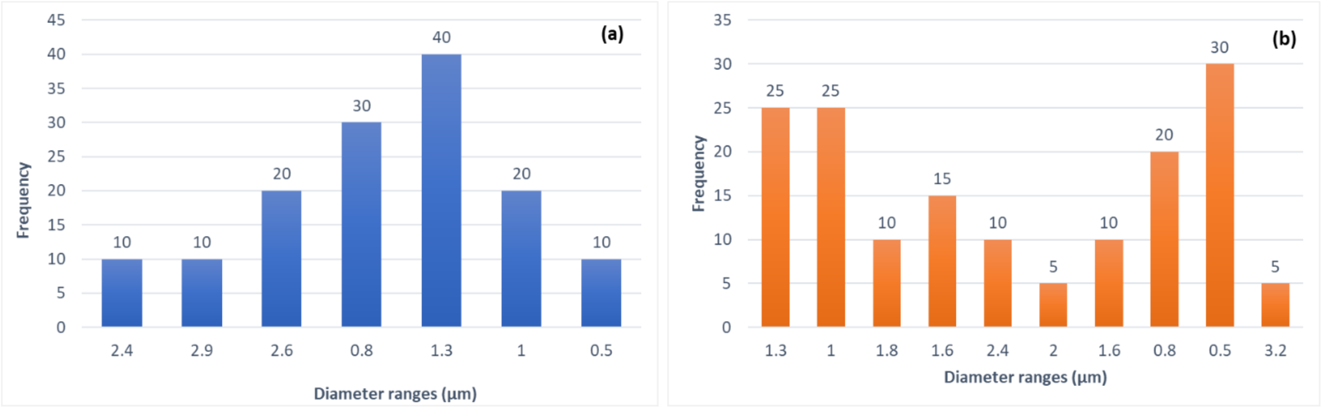

Figure S1: Fiber pore size distributions: a) H_3PO_4-activated carbons; b) KOH-activated carbons.

The H_3PO_4 treatment results in a more centralized particle size distribution with the highest frequency at a diameter of 1.3 µm. In contrast, the KOH treatment shows a broader and more varied distribution with the highest frequencies at diameters of 0.5 µm and 0.8 µm. H_3PO_4 particles predominantly range from 0.5 µm to 2.9 µm, whereas KOH particles have a wider size range extending up to 3.2 µm. Overall, H_3PO_4 produces more uniform particle sizes than the diverse ones from KOH treatment.

Text 1: Dye uptake (mg/g) and dye removal (%) of KOH-activated carbon

Dye uptake (mg/g) is measured by the following formula [6],

$$q_e = \frac{c_o - c_e}{m} \times V$$

where c_0 and c_e are the initial and the equilibrium concentrations (mg/L) of the dye solution, V is the volume of the solution (L), and m is the mass of the adsorbent (g).

Dye removal (%) is measured by the following formula,

$$\text{Dye removal (\%)} = \frac{c_o - c_e}{c_o} \times 100$$

Table S3: Dye uptake (mg/g) and dye removal (%) of KOH-activated carbon derived from raw jute and NaOH-treated jute.

**Dye uptake (mg/g) and dye removal (%) of KOH-activated carbon derived from raw jute**			**Dye uptake (mg/g) and dye removal (%) of KOH-activated carbon derived from NaOH-treated jute**		
**Different concentration** *(ppm)*	**Dye uptake** q_e *(mg/g)*	**Dye removal** *(%)*	**Different concentration** *(ppm)*	**Dye uptake** q_e *(mg/g)*	**Dye removal** *(%)*
10	9.35	93.51	10	6.67	66.79
25	24.12	96.48	25	11.06	44.27
50	22.32	44.65	50	6.20	12.40
100	4.96	4.96	100	6.87	6.87

References

(1) Yousuf, M. R.; Mahnaz, F.; Syeda, S. R. Activated Carbon Fiber from Natural Precursors: A Review of Preparation Methods with Experimental Study on Jute Fiber. *Desalination Water Treat* **2021**, *213*, 441–458. https://doi.org/10.5004/dwt.2021.26731.

(2) Dou, Y.; Liu, X.; Wang, X.; Yu, K.; Liang, C. Jute Fiber Based Micro-Mesoporous Carbon: A Biomass Derived Anode Material with High-Performance for Lithium-Ion Batteries. *Materials Science and Engineering: B* **2021**, *265*. https://doi.org/10.1016/j.mseb.2020.115015.

(3) Cho, D.; Kim, J. M.; Song, I. S.; Hong, I. Effect of Alkali Pre-Treatment of Jute on the Formation of Jute-Based Carbon Fibers. *Mater Lett* **2011**, *65* (10), 1492–1494. https://doi.org/10.1016/j.matlet.2011.02.050.

(4) Basta, A. H.; Fierro, V.; El-Saied, H.; Celzard, A. 2-Steps KOH Activation of Rice Straw: An Efficient Method for Preparing High-Performance Activated Carbons. *Bioresour Technol* **2009**, *100* (17), 3941–3947. https://doi.org/10.1016/j.biortech.2009.02.028.

(5) Cazetta, A. L.; Vargas, A. M. M.; Nogami, E. M.; Kunita, M. H.; Guilherme, M. R.; Martins, A. C.; Silva, T. L.; Moraes, J. C. G.; Almeida, V. C. NaOH-Activated Carbon of High Surface Area Produced from Coconut Shell: Kinetics and Equilibrium Studies from the Methylene Blue Adsorption. *Chemical Engineering Journal* **2011**, *174* (1), 117–125. https://doi.org/10.1016/j.cej.2011.08.058.

(6) Xiong, G.; Wang, B.; You, L.-X.; Ren, B.-Y.; He, Y.-K.; Ding, F.; Dragutan, I.; Dragutan, V.; Sun, Y.-G. *Supplementary Material (ESI) Supporting Information Hypervalent Silicon-Based, Anionic Porous Organic Polymers with Solid Microsphere or Hollow Nanotube Morphologies and Exceptional Capacity for Selective Adsorption of Cationic Dyes*; 2018.
